# Human Immunodeficiency Virus in Adults Undergoing Surgery for Brain Tumors

**DOI:** 10.7759/cureus.26876

**Published:** 2022-07-15

**Authors:** Lauren Harris, Shahinur Rahman, Mohamed Khoudir, Hu Liang Low, Babar Vaqas

**Affiliations:** 1 Department of Neurosurgery, Queen's Hospital, London, GBR

**Keywords:** outcomes, glioma, surgery, brain tumours, human immunodeficiency virus

## Abstract

Introduction

Early diagnosis and treatment of human immunodeficiency virus (HIV) can improve outcomes. HIV prevalence in brain tumor patients and the impact of an HIV diagnosis on patient outcomes are poorly understood.

Materials and methods

This was a prospective study of 100 consecutive brain tumor patients admitted to a Greater London Tertiary Neurosurgical center for surgery between January 2021 and October 2021. All brain tumors were included. All patients have appropriately consented. Blood was tested to detect HIV antibodies and p24 antigen. Outcomes were noted at 30-day postoperative follow-up.

Results

In 100 patients, there was one case of a known HIV-positive, seronegative patient, and no new diagnosis was made, giving a prevalence of 1%. The mean age of patients included was 61.7 ± 13.3, with 57% female. The patient with HIV suffered a postoperative pseudomonas infection, requiring intensive care, additional surgery, and antibiotics. This resulted in an inpatient stay of 55 days - an increase of 274% compared to patients without HIV.

Conclusion

Literature regarding the prevalence of HIV in glioma patients is inconclusive, of low quality, and primarily out-of-date. Our literature search found no similar study of rates of HIV in brain tumor patients in the United Kingdom. The incidence of both HIV and brain tumors, particularly glioblastomas, is low.

## Introduction

Approximately, 101,200 people in the United Kingdom (UK) have human immunodeficiency virus (HIV), 40% of whom live in London, with a prevalence of 2.1 per 1000 in 15-74-year-olds [1]. Currently, 13% are undiagnosed, and there are over 6,000 new diagnoses per year [1]. Of those with known HIV, 96% are on highly active antiretroviral therapy (HAART), and 94% have an undetectable viral load [1]. Testing was done by enzyme-linked immunosorbent assay (ELISA) and western blot antibody detection (at day 30 post-exposure), with a sensitivity of 99.7%, specificity of 98.5%-99.9%, and a cost of £7 [2,3]. The current National Institute for Health and Care Excellence (NICE) guidelines recommend HIV screening as part of routine medical care [2]. In London, many units test all patients requiring blood samples in the emergency department.

The consequence of missing an HIV diagnosis in an area of high prevalence in the general population can lead to potentially avoidable, poor outcomes for the patient. Even one positive diagnosis is cost-effective, given that HAART treatment can lead to a near-normal life. At a national level, in the UK, 0.2% of tests performed for HIV have a positive test result, with this increasing to 0.5% in areas of extremely high prevalence (our catchment area) [2].

HIV prevalence in brain tumor patients and the impact of an HIV diagnosis on individual patient outcomes are poorly understood. This study looks at the prevalence of HIV in all newly diagnosed brain tumor patients, including gliomas, metastasis, and lymphomas. It seeks to address this lack of knowledge by looking directly at the prevalence of HIV in brain tumor patients treated in a single Greater London Tertiary Neurosurgical Center in the UK and its effect on the outcomes.

## Materials and methods

This is a prospective study to assess the rate of HIV infection in 100 consecutive brain tumor patients admitted to Queen’s Hospital, Romford, UK, for a neurosurgical operation between January and October 2021. The catchment area includes Barking, Havering, Redbridge, and Essex. It has a large migrant population, especially from South and Southeast Asia, Eastern Europe, West Indies, and West Africa. It has one of the largest HIV populations in the UK with a prevalence of 5.49 per 1,000 aged 15-59 years and an incidence of 18.4 per 100,000 aged 15+ years [4].

All brain tumors undergoing a neurosurgical procedure were included. This included benign or malignant, primary or metastatic, emergency or elective, debulking or biopsy cases. Adult patients, aged 17 or older, who had the ability to consent to the study, were included. Any brain tumor mimics (e.g., when a suspected tumor was histologically diagnosed as an abscess) were excluded. HIV testing was encouraged as a part of routine clinical care due to the relatively large prevalence in the catchment area [2]. The study was registered with the local review board following the research and development team's approval.

All patients have appropriately consented to the study, prior to testing. Blood samples were collected and tested using fourth-generation tests to detect HIV antibodies and p24 antigen using ELISA and western blot. The microbiology and infectious disease team provided support to this study, offering the appropriate counseling and treatment for any new HIV-positive case identified. Data collection included patient demographics, comorbidities, tumor type, and length of stay. Outcomes were noted at 30-day postoperative follow-up, and data were analyzed using simple, univariate statistics.

## Results

One hundred patients were included in this study, with a mean age of 61.7 ± 13.3 (range: 23-87). The male to female ratio was 0.43:0.57. One patient had known HIV, with an undetectable viral load. No additional patients were found to be positive for HIV. This means the prevalence was 1%.

With regard to risk factors for HIV infection, 91 patients were European, five were Afro-Caribbean, and four were Asians. Ninety patients were born in the UK, four in Asia, two in Africa, two in Europe, one in the Caribbean, and one in Turkey. One patient was an intravenous drug user, one had hepatitis B, and one had tuberculosis. No patients had conditions requiring blood transfusions, and no patients were men who had sex with men.

**Figure 1:**
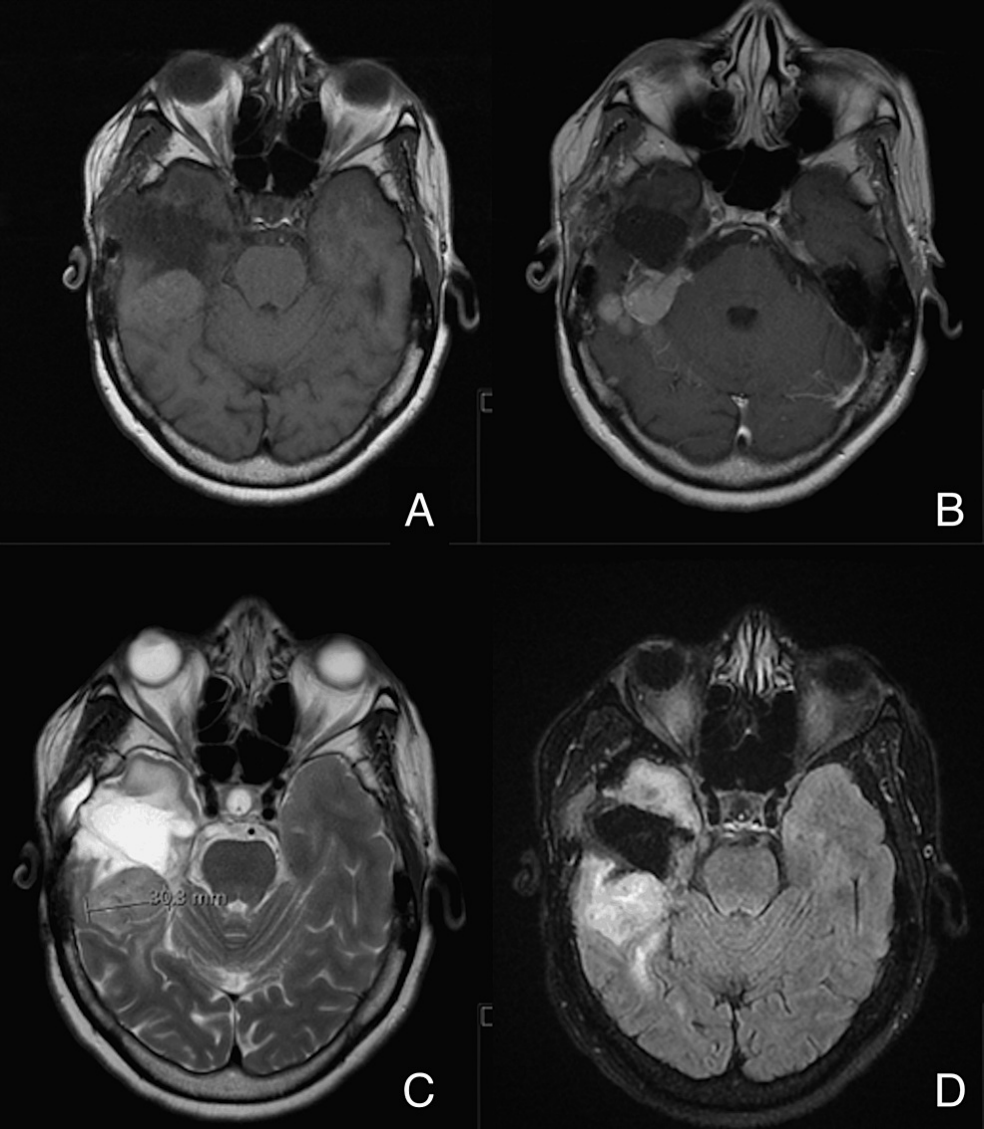
Petrous apex hemangiopericytoma magnetic resonance imaging (MRI) A: T1; B: T1 + contrast; C: T2; D: FLAIR imaging. FLAIR: Fluid-attenuated inversion recovery.

The one positive HIV case was in a 50-year-old female, born in Africa, with long-term well-controlled HIV and a known grade II right petrous apex hemangiopericytoma requiring further surgery (Figure 1).

She originally presented in 2014 with headaches, diplopia, papilledema, and left-sided weakness, which improved with dexamethasone. From the outset, she had known HIV-1, which was well controlled (CD4 count average: 699) on antiviral medication (nevirapine, abacavir, and lamivudine), and her viral load was undetectable. In 2014, a biopsy confirmed a hemangiopericytoma, and she went on to have a resection of 90% of the tumor, with radiation therapy to the residual around the petrous bone and posterior fossa (54 Gy in 30 fractions). From 2017, serial imaging showed an increase in the size of the residual, for which she underwent a further resection in 2021. She was managed with input from her specialist infectious diseases team, who advised that no additional measures were required due to her undetectable viral load.

Initially, she made a good recovery from her surgery. On day 7, she had a partial seizure with discharge from her wound and a C-reactive protein (CRP) of 38. *Pseudomonas aeruginosa* was isolated from the bone flap and subgaleal fluid. The infection resulted in an intensive care admission for seizure control (seven days), a second operation to wash out the infected wound and remove the bone flap, and a long course of antibiotics (seven weeks of intravenous meropenem and gentamicin). Risk factors for infection included revisional surgery, radiotherapy, peri-operative dexamethasone, and immunosuppression. She had an increased inpatient length of stay of 55 days - an increase of 274% compared to patients with an HIV-negative result (mean: 14.7 days ± 23.74). Histology from this second operation showed a grade III malignant hemangiopericytoma (now known as a solitary fibrous tumor).

Twenty-one patients underwent a stereotactic biopsy, and 79 were treated with resection (maximal safe removal of tumor, particularly the enhancing tissue, with preservation of neurological function). The main pathology was glioblastoma, isocitrate dehydrogenase (IDH)-wildtype, World Health Organization (WHO) grade 4 (n = 46), metastatic tumors (n = 20), meningioma (n = 17), and lymphoma (n = 3). A further breakdown of the pathology is shown in Table 1.

**Table 1:** Breakdown of brain tumor diagnosis by pathology

Pathology	Number
Glioblastoma	46
Metastasis	20
Melanoma	6
Lung	5
Breast	5
Colorectal	2
Ovarian	1
Renal	1
Meningioma	17
Other astrocytoma	5
Lymphoma	3
Schwannoma	3
Pituitary adenoma	1
Craniopharyngioma	1
Ependymoma	1
Hemangioblastoma	1
Hemangiopericytoma	1
Chondrosarcoma	1

## Discussion

The prevalence of HIV in 100 consecutive patients undergoing surgery for brain tumors was 1%. In one of the most prevalent catchment areas for HIV in Greater London, we found no new cases of HIV. The only HIV-positive case was in a patient who was known to have seronegative HIV, prior to the diagnosis of her brain tumor. Based on the prevalence of HIV in the catchment area, we would expect one in 182 tests to be positive. Our result of one in 100 is higher than this.

Since its discovery in the 1980s, HIV has increasingly become a chronic condition with a life expectancy similar to the general population due to the advent of therapy with HAART [5]. Most of the existing literature on HIV and brain tumors focus on tumor prevalence in HIV-positive populations rather than the HIV prevalence in tumor patients [6,7]. The use of HAART has dramatically added to the longevity of HIV-positive, seronegative patients, who are now living long enough to develop a range of cancers thought to arise in part due to reduced immune surveillance, direct effects of HIV, or oncogenic viral infection. These include primary CNS lymphoma, Hodgkin's disease, melanoma, lung cancer, renal cancer, brain metastases, and meningiomas [8-10].

High-quality class I/A evidence-based literature on the prevalence of HIV in glioma patients was not identified by this author group. With the use of HAART and the dramatic change in life expectancy for HIV-positive, seronegative patients, much of the literature is out-of-date. Current knowledge of the prevalence of HIV in glioma patients is based on several small case series. Recent studies from Zimbabwe (2018), Kenya (2020), Brazil, and Mexico (2011) suggest a lower prevalence of HIV in glioma patients [11-13]. Other conflicting studies suggest glioblastomas occur at a greater frequency and younger age in the HIV population [10,14]. One Zimbabwean study had a prevalence of HIV in 8.3% of glioma patients versus 14.3% in the general population, with a 42% decrease in gliomas in HIV-positive versus HIV-negative patients [11]. These studies have a different patient demographic to that seen in the UK, with a higher prevalence of HIV. There is no similar study on the rates of HIV in brain tumor patients in the UK. One Polish study has a similar demographic with no cases of HIV identified on screening of 100 patients [15].

In patients with HIV and glioblastoma, HAART is associated with improved survival [16]. Prognosis appears to be predicted by the tumor histology, surgery, or WHO grade and not HIV status [17-19]. No evidence of HIV or Epstein-Barr virus (EBV) infection has been seen in the tumor cells themselves [17]. This has influenced glioma research to use HIV and HAART as starting points. Protease inhibitors have been shown to downregulate vascular endothelial growth factor (VEGF) and hypoxia-inducible-factor (HIF)-1a expression in glioblastoma cells [20]. This has important implications as potential antitumor agents, in particular in combination with other agents.

Although screening all tumor cases being admitted did not pick up a new HIV case, the consequence of missing an HIV diagnosis in an area of high prevalence can lead to poor outcomes, both in terms of HIV treatment and risk management for post-surgical complications of HIV-related infection. The catchment area for this study has a diverse ethnic population; however, the patients in our study were predominantly European- and UK-born. This is likely to indicate a demographic of the disease itself rather than selection bias as the incidence of gliomas is higher in Europeans [21]. Screening may be more effective in high throughput settings such as the emergency department, or community settings, rather than tertiary specialties such as neuro-oncology with a super selected patient base.

There are several limitations to this study. It is a single-center study and only includes patients undergoing surgical intervention for tumors; hence, patients treated non-surgically or on a palliative pathway were excluded. This study did not include patients referred with suspected tumors who were found to have tumor mimics including abscesses (e.g., toxoplasmosis) or other intracranial pathology (e.g., progressive multifocal leukoencephalopathy) associated with HIV. The sample size was only 100, which is inadequately powered for external validity, particularly given the 5.49 per 1,000 prevalence of HIV in the catchment area.

As only one patient was HIV positive and seronegative, no firm conclusions can be made regarding the impact of HIV on brain tumor outcomes, although our single patient had an increased length of stay compared to non-HIV patients. This suggests that it is important to identify brain tumor patients with HIV as they may have a more complex inpatient stay. In addition, this patient had a petrous apex hemangiopericytoma, an uncommon entity in itself, rather than a more common lesion seen in HIV (e.g., CNS lymphoma), limiting generalizations. To address some of these concerns, we are working in conjunction with our infectious disease team to prospectively look at the incidence of brain tumors in known HIV-positive cases.

## Conclusions

No similar study has been performed in the UK. The impact of an HIV diagnosis on brain tumor patient outcomes remains poorly understood. Due to the methodological difficulties when looking at two rare diagnoses, a large multicenter study or national registry of all brain tumor patients is recommended. This would allow a more accurate estimate of HIV prevalence and assess the impact this has on their care and survival. It may also provide epidemiological data to guide the repurposing of antiretroviral drugs for brain tumor treatment.
